# Physical Fitness and Anthropometric Measures of Young Brazilian Judo and Wrestling Athletes and Its Relations to Cardiorespiratory Fitness

**DOI:** 10.3390/sports7020038

**Published:** 2019-02-12

**Authors:** Vitor Marques, Victor Coswig, Ricardo Viana, Acácia Leal, Fagner Alves, Ana Alves, Gabriela Teles, Carlos Vieira, Maria Silva, Douglas Santos, Paulo Gentil

**Affiliations:** 1 Faculty of Physical Education and Dance, Federal University of Goiás, Goiânia 74690-900, Brazil; vitor_alvesmarques@hotmail.com (V.M.); acaciagflealfisio@gmail.com (A.L.); fagnermedeiros10@hotmail.com (F.A.); anagabriela_alves@hotmail.com (A.A.); gabrielaef.ufg@hotmail.com (G.T.); vieiraca11@gmail.com (C.V.); maria2593857@hotmail.com (M.S.); funnyeduca@hotmail.com (D.S.); paulogentil@hotmail.com (P.G.); 2 Faculty of Physical Education, Federal University of Pará, Castanhal 68746-360, Brazil; vcoswig@gmail.com; 3 College of Physical Education, State University of Bahia, Teixeira de Freitas 45992-255, Brazil

**Keywords:** conditioning, fighters, profile

## Abstract

This study aimed to compare the anthropometric profile and physical fitness of young judo and wrestling athletes. Twenty-four young athletes (judo (n = 13) and wrestling (n = 11)) participated in this study. The first visit involved anthropometric and flexibility evaluation, abdominal endurance test, upper limbs resistance and cardiorespiratory test. After 48 h, horizontal jump test (HJT), vertical jump test (VJT), medicine ball throw test (MBT), chin-up test (CUT), chin-up isometric test (CUIT) and the anaerobic resistance test were performed. Judo athletes presented greater values for body mass (*p* = 0.020), height *(p* = 0.010), and body mass index (*p* = 0.026) than wrestlers. Judo athletes also performed better for abdominal endurance (*p* = 0.044), upper limb resistance tests (*p* < 0.001), VJT (*p* = 0.022) and MBT (*p* = 0.023) than wrestling athletes. These results suggest that young judo athletes presented a higher performance in abdominal endurance, upper limbs resistance, HJT, VJT and MBT than wrestling athletes, suggesting that strength and conditioning are related to modality specificity.

## 1. Introduction

Among the combat sports, judo and wrestling are the most represented in the Olympic Games and both distribute many medals in sporting events [1]. Judo is mainly characterised by stand-up techniques aiming to throw or take down the opponent [2,3], while wrestlers aim to physically dominate the opponents and establish control [3,4]. Both are grappling modalities in which athletic performance is influenced by muscular strength, anaerobic power, muscular endurance, aerobic power, flexibility and technical ability [3,4,5], all of which are essential for competitive success [6,7,8].

The technical-tactical training provides support for the adequate development of aerobic endurance, power, anaerobic capacity, strength, speed and flexibility [9,10]. In fact, due to the high-intensity intermittent nature of combat sports, its practice has been suggested as a means to improve fitness [9,11,12,13,14]. Anaerobic systems seem to be the main energy sources for determinant actions (i.e., takedowns and submissions) [11,15]; however, as the match goes on, the aerobic contribution becomes predominant, even if only efforts are considered [11]. Furthermore, it is important to ensure that the athletes’ aerobic system is sufficiently developed for training and competition, since aerobic power enables the maintenance of high intensity activities during the fight, which might allow individuals to maintain high performance for a longer period [10]. The physiological demand for combat sports is very high. Winning depends on the strength and power of the attacks and movements against the opponent. High levels of muscular endurance and ability to maintain high levels of muscle power, strength and speed in combat can contribute to the performance and efficiency of proper motor gesture, thus helping to improve the technical-tactical performance of athletes [9,10].

Many studies have measured parameters of physical performance in both judo and wrestling [16,17,18]. Although there are studies comparing the physical aspects of the two modalities, there is still a gap regarding their physiological aspects. Therefore, this work can help coaches to better plan training. Whilst judo and wrestling are grappling modalities, they are differently categorized as Belt/Jacket and Freestyle [19] respectively, which, in addition to many specificities, might bring different physiological demands and result in different adaptations [20]. Those differences could be related to clothing, duration of the match, and technical-tactical aspects.

In addition, the anthropometric profile has been investigated in combat sports, due to its relationship with physical and technical aspects [6]. The profile of combat athletes changes according to their category, but some valences (e.g., speed, power and strength) correlate with the anthropometric evaluations which are fundamental for better performance during the fights [14]. Indeed, these anthropometric measures are especially relevant for coaches and trainers of young athletes, since it could help in talent identification and selection [15].

However, despite the fact that both judo and wrestling are widely popular and have received attention among Olympic combat sports science in recent years [21], direct comparisons of the physiological profile of athletes from different fighting modalities are scarce [10]. In this context, the objective of the present study was to compare the anthropometric profile and physical fitness of young judo and wrestling athletes.

## 2. Materials and Methods

### 2.1. Experimental Approach to the Problem

Each volunteer reported to the laboratory on two separate occasions. During the first visit, the evaluations involved sociodemographic data, anthropometric measures, flexibility, abdominal endurance, upper limb endurance and cardiorespiratory fitness. In the second visit, power tests (horizontal jump, vertical jump and medicine ball throw test), specific strength tests (fixed bar and fixed bar in isometry) and the anaerobic resistance test were performed. The visits were separated by 48 h and occurred at the same time of day. All participants were instructed to abstain from caffeine, alcohol and strenuous physical activity for 48 h before each test day. The temperature in the testing laboratory ranged from 21 to 23 °C for all trials.

### 2.2. Subjects

Thirteen junior judo athletes (16.4 ± 2.5 years; 66.4 ± 12.1 kg; 1.66 ± 0.07 m) and eleven junior wrestling athletes (15.6 ± 2.6 years; 52.4 ± 15.5 kg; 1.58 ± 0.08 m) participated in the study. The athletes were light weight (judo) or half extra light weight (wrestling), judo athletes had a practice time of 3.2 ± 0.05 years (minimum and maximum training time of 12 and 36 months, respectively) and were between the orange and brown belts, while wrestling athletes had a practice time of 2.8 ± 0.04 years (minimum and maximum training time of 12 and 36 months, respectively). They trained five times a week, with the emphasis on physical work twice a week, and the emphasis on technical work on the other days. Tests were performed between February and March 2016. All athletes were in the pre-competition period, focusing on the national competition of their respective modalities. The inclusion criteria were: (i) To be affiliated with the State Federations of Wrestling or Judo and (ii) to be training to compete. Those athletes who presented some type of functional limitation due to injury or illness were excluded from the study. All participants were informed of the potential risks and benefits of the study and a parent or guardian provided written informed consent. All experimental procedures were approved by the University Ethics Committee (Approval n° 1.645.086) and conformed to the principles outlined in the Declaration of Helsinki. 

### 2.3. Procedures

#### 2.3.1. Anthropometric Evaluation

Body mass was measured by a digital scale (Filizola, Personal 7708, São Paulo, Brazil) and height was measured by a portable stadiometer (Seca, 213, Cotia, Brazil) according to the procedures described by Lohman et al. [22]. The wingspan was measured using a tape measure (TR4012, Sanny, São Paulo, Brazil) as the distance from the right middle finger to the left middle finger with the volunteer standing upright and the arms abducted at 90° with the trunk, elbows extended and forearms supinated, according to the procedures described by Mackenzie [23].

#### 2.3.2. Flexibility Assessment

Flexibility was assessed by the sit and reach test [24] using a Wells Bench (BW2005, Sanny, São Paulo, Brazil) and adopting the procedures established by Wells and Dillon [24]. The flexibility test is important for judo athletes as it helps in injury prevention and assists with strength and power during the fight [25,26].

#### 2.3.3. Abdominal and Upper Limb Endurance

Abdominal endurance was evaluated according to the test described by Mackenzie [23]. The athlete was in the supine position, with their arms crossed and knees flexed, and the athletes made the complete movement [23]. To evaluate the upper limbs, the push-up test was performed. During the test, the volunteers positioned themselves in the supine position, with their hands resting on the ground and fingers facing forward. Then, the volunteers flexed the elbows to approximately 90 degrees and extended them again. Only repetitions with complete range of motion were counted [23]. The resistance test of upper limbs is important because it helps fighters to perform blows and assess the grip of his opponent.

#### 2.3.4. Cardiorespiratory Fitness

Cardiorespiratory fitness was assessed by an incremental ergospirometric test on a motorised treadmill (Centurion, 200, Micromed 2000, Brazil) coupled to a portable computer. Participants remained at rest in the orthostatic position for 3 min pre-exercise, followed by a 2-min warm up at 5 km/h. Speed was increased by 1 km/h every minute until exhaustion. After exhaustion, active recovery was performed for 2 min at 2 km/h and the participant then sat for 4 min. The criteria used for test interruption were: (i) Incapacity of the participant to perform the exercise; (ii) accentuated increase in systolic arterial pressure (reaching values greater than 200 mmHg); and (iii) reaching maximum age-predicted heart rate (HR) or (iv) respiratory exchange ratio >1.15 [27].

HR was continuously monitored using an HR monitor (Polar Electronics, V800, Kempele, Finland). The expired air was continuously measured breath-by-breath using a portable gas analyser (Cortex, Metalyzer II, Rome, Italy). Peak treadmill speed (*v*$\dot{V}$O_2_peak) was defined as the last achieved running speed sustained for at least 30 s. $\dot{V}$O_2_peak was defined as the highest 10-s averaged $\dot{V}$O_2_ value with inclusion criteria consistent with conventional guidelines for $\dot{V}$O_2_peak [28].

#### 2.3.5. Horizontal Jump, Vertical Jump and Medicine Ball Throw Test

Participants started the horizontal jump test (HJT) with both feet parallel behind a marked start line; participants were instructed to cover the greatest horizontal distance possible using both feet and swinging their arms. The distance covered by the jump was determined from the start line to the heel that landed closest to the start line using a floor mounted measure tape. Three attempts were performed with one min of rest separating each set [23] and the highest value was used in the analysis.

Vertical jump test (VJT) involved measures of the difference between the standing reach and the highest height reached during a vertical jump. To do this test, the athletes chalked the end of their fingers. The athlete stood side onto the wall, keeping both heels on the ground, reached up as high as possible with one hand and marked the wall with the tips of their fingers. The athlete then jumped as high as possible and marked the wall again. The vertical displacement was then calculated on a tape mounted on the wall. Each athlete performed three jumps with one minute of rest in between, and the best jump was selected for analysis [23,29,30].

The medicine ball throw test (MBT) is a common measure of upper-body explosive power [31]. It is conducted using a standard 3 kg medicine ball. Participants were seated on the ground with their legs fully extended and back against the wall. The medicine ball was held with both hands against the chest and the forearms were positioned parallel to the ground. Participants were instructed to throw the medicine ball as far as they could while keeping their back against the wall. The best performance of three trials was recorded.

All of these tests aim to evaluate muscular power, a fundamental variable for combat athletes because it is directly associated with the speed of reaction and the speed of execution of the blows during the fight.

#### 2.3.6. Specific Strength Tests

Athletes’ strength-endurance was assessed using maximum repetitions chin up test (CUT), where the athletes were encouraged to perform as many repetitions as possible [23,32]. Fifteen minutes after the CUT, the chin up isometric test (CUIT) was performed. In CUIT, the athletes were asked to keep their elbows flexed with their chin above the hands for as long as possible and the holding time was recorded. The judo athletes used judogi because of the specificity of the sport. The test was interrupted as soon as athletes were unable to maintain the initial isometric position [23]. Strength testing is essential as it assesses the athlete’s ability to perform the strokes more safely, bring greater dominance over the opponent, and increase decision-making power during the fight.

#### 2.3.7. Anaerobic Capacity Test

Each athlete underwent an anaerobic resistance test on a motorised treadmill (Centurion, 200, Micromed 2000, Brazil). The test consisted of a 10-min warm-up involving five bouts of 60 s at 10 km·h^−1^ interspaced by 60 s at 6 km·h^−1^ (60:60 s). Then, the speed was increased to 13 km·h^−1^ with a 20% slope and maintained until exhaustion [33]. During the test, athletes were verbally encouraged to exercise for as long as possible. Time of test and HR were recorded using an HR-monitor (Polar Electronics, V800, Kempele, Finland).

### 2.4. Statistical Analysis

The normality of the data was evaluated by the Shapiro-Wilk test. Data were expressed as mean and standard deviation (SD). The categorical data were presented as a relative frequency. Pearson’s Chi-squared was used to compare BMI, socio demographic variables and lifestyle data among judo and wrestling athletes. The Student’s *t*-test for independent samples (data with normal distribution) or Mann-Whitney test (data with non-normal distribution) were used to compare the anthropometric, physical and ventilatory variables among the athletes of both modalities. Pearson’s and Spearman’s correlation coefficient was used to determine correlations between the variables with normal or non-normal distribution, respectively. Correlations below 0.49 were described as “poor,” from 0.50 to 0.69 as “moderate,” and 0.70 to 0.89 as “high,” and from 0.9 and above as “very high” [34]. Statistical Package for the Social Science software (version 21.0, IBM Corp., Armonk, NY, USA) was used and *p* < 0.05 was considered significant.

## 3. Results

There was no difference between groups for age (judo athletes: 16.4 ± 2.5 years vs. wrestling athletes: 15.6 ± 2.6 years, *p* = 0.303). Judo athletes presented significantly greater body mass (*p* = 0.020), height (*p* = 0.010) and BMI (*p* = 0.026) than wrestling athletes. No significant difference was found in the cardiorespiratory and performance variables between judo and wrestling athletes (*p* > 0.05). Judokas presented higher abdominal endurance (*p* = 0.044), upper limb endurance (*p* < 0.001), VJT (*p* = 0.022) and MBT (*p* = 0.023) than wrestlers. However, when relativized to body mass, the values VJT (*p* = 0.617) and MBT (*p* = 0.608) did not show significant differences between modalities (*p* > 0.05). Table 1 shows additional information about anthropometric profile, performance exercise cardiorespiratory and physical performance of the athletes.

A significant positive correlation was found between judo athletes’ age and upper limb endurance (*r* = 0.60 (moderate); *p* = 0.030). No significant correlation was found between wrestling athletes’ age and any variable (*p* > 0.05). A significant positive correlation was found between judo athletes’ $\dot{V}$O_2_max and their performance in the HJT (r = 0.84 (high); *p* < 0.001), VJT (r = 0.58 (moderate); *p* = 0.036), CUT (r = 0.75 (high); *p* = 0.003) and MBT (*r* = 0.61 (moderate); *p* = 0.027). A significant positive correlation was found between wrestling athletes $\dot{V}$O_2_max and their performance in the CUT (*r* = 0.61 (moderate); *p* = 0.045), CUIT (*r* = 0.81 (high); *p* = 0.002), anaerobic resistance (*r* = 0.67 (moderate); *p* = 0.023) and abdominal endurance (*r* = 0.62 (moderate); *p* = 0.043).

Judo athletes presented a significant positive correlation between height and performance in the VJT (*r* = 0.60 (moderate); *p* = 0.030), and between VJT (*r* = 0.63 (moderate); *p* = 0.063) and body mass and wingspan (r = 0.66 (moderate); *p* = 0.013). Also, significant positive correlations were found between performance in the MBT and height and body mass (r = 0.71 (high); *p* = 0.007 and *r* = 0.60 (moderate); *p* = 0.032, respectively). Wrestling athletes only presented a significant positive correlation between performance in the HJT and height (*r* = 0.73 (high); *p* = 0.010). Table 2 shows additional information about the correlation between physical performance and anthropometric profile of judo and wrestling athletes.

## 4. Discussion

The objective of the present study was to compare the anthropometric profile and physical fitness of young judo and wrestling athletes. The main results of the present study were that: (i) Judo and wrestling athletes presented different anthropometric characteristics (body mass, height and BMI); (ii) judo and wrestling athletes presented similar aerobic power; (iii) judokas had a better performance than wrestlers in four (abdominal endurance, upper limbs resistance, VJT, and MBT) of the nine tests performed; and (iv) there was a significant correlation between $\dot{V}$O_2_max and physical performance of wrestling and judo athletes with some of the tests performed.

Our results are contrary to those of Iwai et al. [20], who did not find any differences in the height and body mass between judo and wrestling athletes. These inconsistencies could be due to the category of athletes since the wrestling athletes of the present study are in a lower category than judo athletes. The high body mass variability among judo (66.4 ± 12.1 kg) and wrestling (52.4 ± 15.5 kg) athletes are described by Franchini et al. [35]. Indeed, wrestlers showed lower height and body mass than previously described by other studies [36,37]. Nonetheless, our findings suggested that it is related to physical fitness differences between judo and wrestlers, since the judo athletes presented a significant moderate positive correlation between body mass and VJT and MBT, and the wrestlers presented a significant moderate positive correlation between height and performance in the HJT. Those results are in agreement with what would be expected from body mass and height, but not from body fat percentage [38].

It is known that wrestling and judo are intermittent and high-intensity combat sports which are related to a constant overload of muscles and joints, especially the shoulder, trunk and hip [9]. Therefore, improvements in strength and fitness are needed to avoid injury and improve performance during fights. In the present study, judo athletes performed better in the abdominal resistance test, upper limb strength test, VJT and MBT than wrestling athletes. This shows us a tendency for judo athletes to perform better and lower chances of injury than wrestling athlete modalities. Despite previous studies that have reported that judo athletes present greater performance in abdominal endurance, upper limb endurance, trunk flexibility, CUT and strength [9,25], no difference was found in the present study in the flexibility between judo and wrestling athletes. Similar results on flexibility were reported by Pion et al. [39], in which the hamstring flexibility of adolescents from several sports modalities was compared. The results showed that judo athletes presented similar values (37.8 ± 6.8 cm) to those found in our study (35.0 ± 6.5 cm). On the other hand, Nikkoie et al. [40] found greater values for the seat and reach test in junior wrestlers (37.0 ± 5.3 cm) than those presented here (32.7 ± 10.5 cm). 

A higher handgrip strength is expected from grappling combat sports, as reported by Sterkowicz, et al. [41], who reported greater handgrip strength in 13 judo athletes when compared to 19 students of the same age and body mass (47.6 ± 9.3 vs. 45.2 ± 5.5 kgf). In addition, due to judogi usage, higher dynamic handgrip and isometric strength and endurance would be expected in judokas than wrestlers [41]; however, no differences between them were found in the present study.

Our findings showed that power tests (HJT, VJT and MBT) of the judo athletes presented a significant positive correlation with aerobic power. Similar results were found by Drid et al. [42], in which international level judo athletes with greater physical fitness, presented better performance in power tests (HJT: 2.87 ± 0.12 m; VJT: 3.2 ± 0.1 m) than sub-elite judo athletes (HJT: 2.6 ± 0.5 m; VJT: 3.1 ± 0.04 m).

In our study, judo athletes presented $\dot{V}$O_2_ values of (37.3 ± 7.2 mL/kg/min) lower than reported in the study by Drid et al. [42], which could be expected, due to the lower competitive level and age differences. Regarding the HJT and VJT tests, the athletes in this study had superior values to those reported by Drid et al. [42], who evaluated adult athletes. Whilst it is not possible to establish a causal relationship between the tests, these results might be explained by the fact that the aerobic-anaerobic transition zone is key to developing the aerobic power and aerobic capacity [43] while judo requires a high level of energy production from both systems [43]. Confirming this, Laskowski et al. [44] reported that judo training improves both aerobic and anaerobic performance. Mirzaei et al. [36] and Passelergue et al. [7] suggest that aerobic power must be developed for optimal levels to allow the athlete to maintain high activity level during the fight without excessive fatigue. We found a significant positive correlation between aerobic power of the wrestling athletes and the strength and muscle endurance tests (CUT, CUIT, anaerobic resistance and abdominal endurance). There were also correlations between aerobic power and strength in the CUT of judo athletes. In this sense, while muscle strength might be important, mainly in the actions of attack and defence to the opponent [41], anaerobic endurance tests are important for assessing extreme fatigue conditions during combat [45].

## 5. Study Limitations

Some limitations should be considered while interpreting our findings. First, technical-tactical sessions in combat sports could be widely different between coaches or regions; however, it could be argued that most coaches follow already established patterns for each modality, which has both positive and negative effects. Second, a larger sample could strengthen our findings; however, it would probably make it easier to statistically reject the null hypothesis. Third, additional measures of body composition were not performed. It could help to explain correlations between anthropometrics and performance markers. Finally, practice time showed near significant differences between groups. Despite some arguing that it would influence physical findings and explain higher values in judokas, we believe that, in practice, both groups are classified as “trained” and differences would be smaller if only practice time was considered.

## 6. Conclusions

Our findings suggest that judo athletes showed higher abdominal and upper limb endurance, vertical jumping and medicine ball throwing. However, when body mass was considered, vertical jumping and medicine ball throw were similar between modalities. In addition, there is a positive and significant correlation between vertical jump with body mass, wingspan and height. The medicine ball throw had a significant positive correlation between height and body mass in judo athletes. Wrestling athletes, on the other hand, demonstrated a positive correlation between horizontal jump and height. The most important finding of our study was that strength and conditioning are related to modality specificity. As previously mentioned, some researchers have suggested that the practice of combat sports by itself should be considered when physical training is planned. To illustrate that, in a training week where an athlete would perform alternating tactical-technical sessions and conditioning sessions, the coach could: (i) Maintain high demand tactical training and reduce conditioning sessions or; (ii) maintain the number of sessions, but reduce the physical demand of tactical training. Both choices would permit strength and conditioning improvements and reduce non-functional overreaching or overtraining, especially in judo. The extra conditioning session should possibly be considered more relevant for wrestling therefore. 

## Figures and Tables

**Table 1:** Physical characteristics, cardiorespiratory exercise performance and physical performance of the judo and wrestling athletes.

Variables	Judo (n = 13)	Wrestling (n = 11)	*p* *
Body mass (kg)	66.4 ± 12.1	52.4 ± 15.5	0.020 *
Height (m)	1.66 ± 0.07	1.58 ± 0.08	0.010 *
BMI (kg/m^2^)	23.9 ± 3.1	20.7 ± 4.8	0.026 *
Wingspan (m)	1.70 ± 0.09	1.62 ± 0.12	0.059
Practice time (years)	3.2 ± 0.05	2.8 ± 0.04	0.079
$\dot{V}$O_2_peak (mL/kg/min)	37.3 ± 7.2	37.9 ± 9.5	0.850
HRpeak (bpm)	180 ± 17	175 ± 11	0.446
*v*$\dot{V}$O_2_peak (km/h)	13.3 ± 2.4	12.9 ± 1.3	0.631
Trunk flexibility (cm)	35.0 ± 6.5	32.7 ± 10.5	0.510
Abdominal endurance (reps)	42.0 ± 11.6	31.7 ± 11.2	0.044 *
Upper limbs endurance (reps)	40.5 ± 16.1	15.1 ± 10.3	<0.001 *
HJT (m)	2.11 ± 0.46	1.80 ± 0.32	0.077
VJT (cm)	45.9 ± 12.4	35.5 ± 7.1	0.022 *
MBT (m)	3.01 ± 0.71	2.25 ± 0.82	0.023 *
CUT (reps)	6.5 ± 5.8	3.0 ± 3.6	0.207
CUIT (s)	34.3 ± 21.5	24.1 ± 21.2	0.256
Anaerobic resistance (s)	41.4 ± 14.2	35.8 ± 22.1	0.465

Data are presented by mean ± standard deviation. * Significant difference between judo and wrestling group. $\dot{V}$O_2_peak, peak oxygen uptake; HR peak, peak heart rate *v*$\dot{V}$O_2_peak, velocity associated at peak oxygen uptake; HJT, horizontal jump test; VJT, vertical jump test; CUT, Chin Up Test; CUIT, chin up isometric test; MBT, medicine ball throw test.

**Table 2:** Correlation between physical performance and anthropometric profile of judo and wrestling athletes.

Variables	Judo (n = 13)				Wrestling (n = 11)			
	Height (m)	BM (kg)	BMI (kg/m^2^)	Wing (m)	Height (m)	BM (kg)	BMI (kg/m^2^)	Wing (m)
Trunk flexibility (cm)	0.01	0.40	0.52	0.11	0.38	0.11	0.04	0.26
Abdominal endurance (reps)	0.21	−0.23	0.44	−0.03	−0.10	−0.07	−0.03	−0.36
Upper limbs endurance (reps)	0.13	0.08	0.04	0.10	0.38	0.09	−0.01	0.12
HJT (m)	0.47	0.39	0.23	0.39	0.74 *	0.36	0.19	0.50
VJT (cm)	0.60 *	0.63 *	0.50	0.67 *	−0.12	−0.39	−0.43	−0.36
MBT (m)	0.71 *	0.59 *	0.35	0.54	0.54	0.49	0.41	0.44
CUT (reps)	0.38	0.03	−0.18	0.22	0.23	−0.16	−0.27	−0.09
CUIT (s)	0.38	0.12	−0.05	0.25	0.30	−0.17	−0.29	−0.02
Anaerobic resistance (s)	0.09	−0.24	−0.36	−0.02	−0.24	−0.33	−0.30	−0.74

HJT, horizontal jump test; VJT, vertical jump test; CUT, Chin Up Test; CUIT, chin up isometric test; MBT, medicine ball throw test. Data are presented by mean ± standard deviation. * *p* < 0.05 between judo and wrestling group.
